# An advanced fragment analysis-based individualized subtype classification of pediatric acute lymphoblastic leukemia

**DOI:** 10.1038/srep12435

**Published:** 2015-07-21

**Authors:** Han Zhang, Hao Cheng, Qingqing Wang, Xianping Zeng, Yanfen Chen, Jin Yan, Yanran Sun, Xiaoxi Zhao, Weijing Li, Chao Gao, Wenyu Gong, Bei Li, Ruidong Zhang, Li Nan, Yong Wu, Shilai Bao, Jing-Dong J. Han, Huyong Zheng

**Affiliations:** 1 Beijing Key Laboratory of Pediatric Hematology Oncology, National Key Discipline of Pediatrics, Ministry of Education, Key Laboratory of Major Diseases in Children, Ministry of Education, Hematology Oncology Center, Beijing Children’s Hospital, Capital Medical University. Beijing, China; 2 CAS Key Laboratory of Computational Biology, CAS-MPG Partner Institute for Computational Biology, Shanghai Institute for Biological Sciences, Chinese Academy of Sciences. Shanghai, China; 3 Ningbo Health Gene Technologies Ltd. Ningbo, Zhejiang, China; 4 State Key Laboratory of Molecular Developmental Biology, Institute of Genetics and Developmental Biology, Chinese Academy of Sciences, Beijing, China; 5 Graduate School of Chinese Academy of Sciences, Beijing, China

## Abstract

Pediatric acute lymphoblastic leukemia (ALL) is the most common neoplasm and one of the primary causes of death in children. Its treatment is highly dependent on the correct classification of subtype. Previously, we developed a microarray-based subtype classifier based on the relative expression levels of 62 marker genes, which can predict 7 different ALL subtypes with an accuracy as high as 97% in completely independent samples. Because the classifier is based on gene expression rank values rather than actual values, the classifier enables an individualized diagnosis, without the need to reference the background distribution of the marker genes in a large number of other samples, and also enables cross platform application. Here, we demonstrate that the classifier can be extended from a microarray-based technology to a multiplex qPCR-based technology using the same set of marker genes as the advanced fragment analysis (AFA). Compared to microarray assays, the new assay system makes the convenient, low cost and individualized subtype diagnosis of pediatric ALL a reality and is clinically applicable, particularly in developing countries.

Leukemia is the most common neoplasm and one of the primary causes of death in children. Each year in China, nearly 15,000 children are newly diagnosed with leukemia. Acute lymphoblastic leukemia (ALL) accounts for 75–80% of pediatric leukemia, and since the 1980s, treatment has achieved a greater than 85% survival rate through standardized anti-leukemic protocols and multi-chemotherapy regimens^1^,^2^,^3^,^4^. However, clinical studies indicate that more intensive therapy is unlikely to raise the cure rate substantially and will instead increase therapy-related mortality and the risk of life-threatening late sequelae such as secondary cancers^5^. One of the main obstacles to improving treatment is the lack of facilities for reliable risk classification, particularly in developing countries, making it difficult to perform the appropriate stratified schemes.

The development of leukemia has been recognized as a process involving multiple genes and signal transduction pathways^6^. Current risk classification incorporates pretreatment clinical characteristics (age, white blood cell count, response to induction therapy and presence of extramedullary disease), the combined evaluation of morphology, immunology, cytogenetics and molecular biology (MICM), and measures of minimal residual disease (MRD) to classify children with ALL into different risk categories. However, most of these advanced technologies are only offered in a few major medical centers in China. Many hospitals do not have these technologies, particularly in the countryside, which is home to the majority of the Chinese population. Moreover, some of the technologies such as cytogenetics and molecular biology are labor-intensive and time-consuming. Such results are usually obtained after 1–2 weeks from different clinical labs, which may delay the stratified therapy. Far more importantly, nearly 50% of children with ALL lack any previously known cytogenetic or molecular lesion. Although the patients share cytogenetic and molecular biological disorders and receive the same chemotherapy regimens, completely different responses to the therapy may arise. These differences indicate that ALL patients with the same risk classification may have underlying molecular differences. To summarize, the traditional risk classification method is clearly inadequate for elucidating the complicated subtypes of leukemia, and it is imperative to explore a novel classification method that can be implemented at the initial diagnosis of pediatric ALL.

High-throughput technologies such as microarrays have spurred the search for gene expression-based markers for ALL classification. By comparing genome-wide gene expression among the subtypes of ALL, between 80 and 300 genes have been identified as marker genes necessary to discriminate among the subtypes^7^,^8^,^9^,^10^,^11^,^12^,^13^. In recent years, a substantial number of studies revealed either novel subtypes or biomarkers for childhood ALL based on high-throughput technologies^14^,^15^,^16^,^17^. In one of our previous studies combining microarray data from 535 Caucasian pediatric ALL samples, we identified 62 marker probe sets (mapped to 61 ENTREZ genes), which could classify ALL into 6 known subtypes, including t(12;21) (*ETV6-RUNX1*), t(9;22) (*BCR-ABL1*), t(1;19) (*TCF3-PBX1*), t(4;11) *MLL* rearrangement, T-ALL, hyperdiploid with more than 50 chromosomes (hyperdiploid >50), and 1 unknown (Others) subtype. The prediction accuracy for each subtype was greater than 97% using an independent microarray dataset from 100 Chinese pediatric ALL patients^18^.

A microarray-based classification method can predict ALL subtypes with high accuracy but is also labor-intensive and time-consuming with low sample throughput and high reagent cost. Microarray results usually require at least 2 weeks and cost approximately 500 US dollars. Although some commercial microarray can be finished within 48–72 hours, it usually takes as long as two weeks, which may lead to missed treatment opportunities. Furthermore, many families in China cannot afford the high cost.

The advanced fragment analysis (AFA)-based technique provides an alternative method for high-throughput multiplexed quantitative gene expression. AFA integrates multiplexed reverse transcription PCR (RT-PCR) and capillary electrophoresis to offer a simple and effective way to analyze dozens of genes in a single tube. With the AFA technology, the turn-around time is dramatically reduced to half a day, and the cost is only 100 US dollars per patient.

In this study, the AFA technology was used to design a multiplex qPCR reaction of 57 marker genes based on our previous results^18^. The classifier we designed has a prediction accuracy of greater than 94% in 160 newly diagnosed patients when using the AFA data. These results demonstrate the bright prospect of using AFA for the accurate diagnosis of pediatric ALL in a reasonable time frame and at an economic cost that is appropriate in developing countries, such as China.

## Methods

### Patient information

A total of 160 children (aged 9 months to 13 years, with a median age of 5 years) diagnosed with ALL between August 2007 and January 2014 were enrolled in this study, which was performed in the Hematology Oncology Center of Beijing Children’s Hospital, Capital Medical University. Informed consent was obtained from all parents or legal guardians. The study was designed in accordance with the Declaration of Helsinki and was approved by the Beijing Children’s Hospital ethics committee prior to its initiation.

All patients were diagnosed with ALL using a combination of MICM classifications. The cytogenetic ALL subtypes were experimentally identified by G-banding karyotype and multiplex nested reverse-transcription-polymerase chain reaction (PCR). We tested for the presence of 45 fusion genes, including *ETV6-RUNX1*, *BCR-ABL1*, *TCF3-PBX1*, *MLL-AF4*, and *SIL-TAL1* (Supplementary Table S1). The patients who were either diploid or hyperdiploid >50 were further identified by a DNA index assay (Cycletest^TM^ Plus DNA Reagent Kit, BD, USA).

The bone marrow (BM) samples from 160 pediatric patients were collected at the time of initial diagnosis (ID), and the patients’ clinical characteristics are described in detail in Supplementary Table S2.

### Primer design

Sixty-two target genes (mapped to 61 ENTREZ genes) were divided into 3 panels, with each panel containing 20 or 21 genes. Each primer consisted of a 3′ gene-specific sequence and a 5′ universal tag sequence. The gene-specific portion of the primer was designed based on the sequence information from NCBI. The common region of all transcript variants of each gene was selected for primer design. Homologous regions prone to mispriming were excluded. Primer pairs were separated by at least one intron on the corresponding genomic DNA, if applicable. The primer sequences were evaluated using BLAST searches to ensure no SNP was present at their 3′ ends and specific amplification of PCR fragments. The gene-specific primers were designed to yield PCR fragments at least 4 base pairs (bp) apart, ranging from 137 bp to 316 bp after a universal tag sequence was added. The forward and reverse universal primer sequences were 5′-AGGTGACACTATAGAATA-3′ and 5′-GTACGACTCACTATAGGGA-3′, respectively.

In addition, each panel was incorporated with 5 reference genes, including *B2M*, *PSMC4* and *GUSB*, as the endogenous reference genes for normalization and calculation of fold change and 2 external synthetic reference controls *Kan*^*R*^ and pcDNA3.1(+) as quality controls for the RT-PCR reaction. *Kan*^*R*^ was used to monitor both RT and PCR steps, while pcDNA3.1(+) was only used to monitor PCR procedure. All primers were purchased from Invitrogen (Shanghai, China).

### AFA-based multiplex assay

For each reaction, 20 ng/μl RNA from each sample was reverse transcribed using the following reaction system: 4 μl of RNA sample (20 ng/μl), 1 μl of RT primer mix (the concentration of each RT primer mix was listed in Supplementary Table S3), 2 μl of 5 × RT buffer [250 mM Tris-HCl (pH 8.3), 375 mM KCl, 15 mM MgCl_2_ and 50 mM DTT], 1 μl of *Kan*^*R*^ (0.1 ng/μl), 1 μl reverse transcriptase (10 units/μl), and 1 μl of DNase/RNase free-H_2_O. RT reactions were performed in a Veriti® Thermal Cycler (Applied Biosystem, USA) using the following reaction program: 48 °C for 1 min, 42 °C for 60 min and 95 °C for 5 min, followed by holding at 4 °C.

An aliquot (4.65 μl) of template cDNA from each RT reaction was transferred to a tube containing the PCR master mix. The PCR master mix incorporates 1 μl of PCR primer mix (the concentration of each PCR primer was 200 nM), 1 μl of CM01, 1 μl of 10 × PCR buffer [100 mM Tris-HCl (pH 8.3), and 500 mM KCl], 0.35 μl of DNA polymerase (2.5 units/μl) and 2 μl of MgCl_2_ (25 mM). CM01 contains the fluorescence-labeled universal forward primer (12 μM), unlabeled universal reverse primer (12 μM), and dNTP (3.5 mM). Multiplex PCR was then performed using a Veriti® Thermal Cycler as follows: 95 °C for 10 min, 35 cycles of 94 °C for 30 sec, 60 °C for 30 sec and 70 °C for 1 min, followed by 70° for 10 min of additional extension, and holding at 4 °C. All reagents in the RT/PCR reactions were provided by Ningbo Health Gene Technologies Ltd (Ningbo, China).

An aliquot (1 μl) of the PCR product for each sample was prepared for capillary electrophoresis by adding 38.8 μl of CEQ Sample Loading Solution (Beckman Coulter, USA) and 0.2 μl of CEQ DNA Size Standard 400 (Beckman Coulter, USA) in a 96-well CEQ electrophoresis plate (Beckman Coulter, USA). The PCR products were analyzed by a Beckman Coulter GeXP Genetic Analysis system (Beckman Coulter, USA) using the separation method of “Frag-3”. The relative quantification of each gene in a sample was determined using standard curve.

### Microarray and AFA data processes

The microarray data from 240 children with an initial diagnosis of ALL, 190 children from the Cooperative ALL Study Group (COALL) and 107 from the Dutch Childhood Oncology Group (DCOG) were downloaded from the GEO database (http://www.ncbi.nlm.nih.gov/geo/; GSE28497, GSE13425 and GSE133512, respectively). The mean value of different probe sets for the same gene was used as the expression value for the gene. Altogether the expression levels of 57 marker genes from 160 clinical samples were obtained from AFA multiplexed assays. Genes that were expressed at low levels and did not exhibit a signal in AFA assays were labeled as zero. Then 57 marker genes’ expression values were sorted from low to high in a patient. Lowly expressed genes have lower rank values and highly expressed genes have higher rank values. Then the rank values instead of the true signal for each gene were used for classification and prediction.

### Support vector machine (SVM) model

The SVM analysis was conducted by using the ‘e1071’ R package (http://www.r-project.org/).

## Results

### Marker gene set minimization and AFA optimization for pediatric ALL subtypes

In our previous study, 62 marker probe sets (mapped to 61 ENTREZ genes) were identified as capable of classifying pediatric ALL based on genome-wide microarray gene expression profiles^18^. Here we examined whether we could use these same genes to develop a subtype classifier based on the AFA multiplexed assay. Among them, two genes (*JUN* and *C10ORF10*) are uni-exonic, and the *IGHD* gene has no reference RNA. To avoid the risk of DNA contamination in the RNA samples, these 3 genes were excluded. Additionally, the *GNPDA1* gene was excluded because its expression level was too low to detect. Consequently, 57 genes (58 primer pairs in total because of a duplicate *PBX1* gene) were selected and randomly divided into 3 panels, with each panel containing 19 or 20 genes. The primers of these genes and the reference controls used in the AFA multiplex qPCR assay are described in Supplementary Table S3.

**Figure 1:**
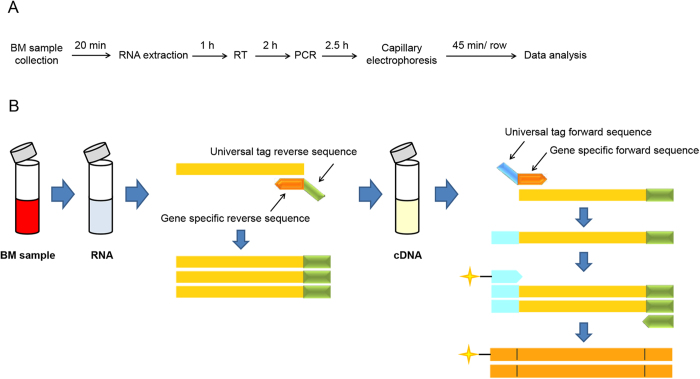
Schematic illustration of the AFA multiplex assay. (**A**) Schematic of the AFA multiplex assay protocol along with the approximate time required for each step. It combines the multiplex reverse transcript (RT), PCR amplification and capillary electrophoresis steps. (**B**) Schematic summarizing the steps of the AFA multiplex assay. Total RNA was extracted from bone marrow samples. Two types of primers were designed: chimeric primers and universal primers. Each chimeric primer consists of a gene-specific sequence at the 3′ end and a universal tag sequence at the 5′ end. The reverse chimeric primers were used to produce the specific cDNA of each gene during reverse transcription from RNA. Both reverse and forward chimeric primers were used at the first stage of PCR amplification. In the second stage, the multiplex amplification was quickly overtaken by the labeled universal primers, which maintain the same amplification efficiency of each gene. Details for each step are provided in the methods section.

**Figure 2:**
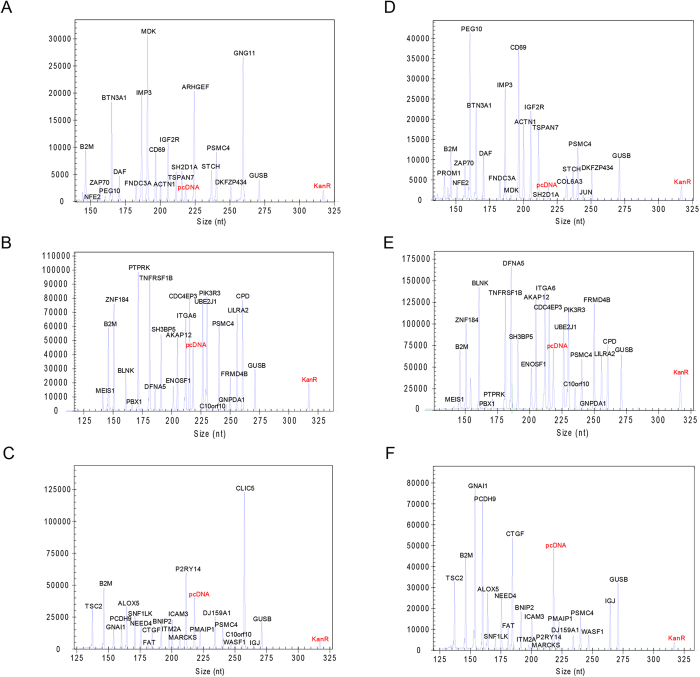
Representative electropherograms corresponding to gene expression profiles generated from *ETV6-RUNX1*-positive and *BCR-ABL1*^-^ positive pediatric ALL RNA samples. *ETV6-RUNX1*-positive RNA sample: (**A**) panel 1; (**B**) panel 2; (**C**) panel 3. *BCR-ABL1*-positive RNA sample: (**D**) panel 1; (**E**) panel 2; (**F**) panel 3. Capillary electrophoresis was performed on a GeXP Genetic Analysis System. Two external controls KanR and pcDNA3.1(+) were highlighted with red colors in each panel. pcDNA represents pcDNA3.1(+).

The specificity of the AFA assay for all marker genes was tested individually in a multiplex assay (data not shown). No mispriming was observed when all pairs of the chimeric primers and the internal control primers were mixed together. The final concentrations of the reverse primers in multiplexed RT-PCR assays were optimized (Supplementary Table S3). The amplicon mixture was analyzed using fluorescence capillary electrophoresis to identify the peak location (gene identity) and peak fluorescence intensity (gene expression level). The schematic protocol of the AFA multiplex assay is illustrated in detail (Fig. 1A,B). Figure 2 shows an example of the 3-panel raw data from the AFA multiplex assay for 2 pediatric ALL patients with different fusion genes. The specific products could be separated clearly from other targets, and different gene expression levels (peak area) were observed.

### AFA multiplex qPCR analysis of pediatric ALL gene expression

In total, 160 pediatric ALL patients with 7 main subtypes were enrolled in this study, including t(12;21) (*ETV6-RUNX1*), t(9;22) (*BCR-ABL1*), t(1;19) (*TCF3-PBX1*), *MLL* rearrangement, T-ALL, hyperdiploid >50, and Others (diploid without chromosomal translocation or fusion gene).

To enable an individualized diagnosis without the background normalization that is needed for routine gene expression analysis, we used the ranks of gene expression values instead of the actual expression values to construct the subtype classifiers^18^. Such a rank-based method provides internal normalization similar to quantile normalization, allowing us to compare marker gene expression across different samples and different platforms without the need for a large panel of additional samples from the same platform to correct for background differences. For each sample assayed in this system, we first sorted the expression levels of the 57 marker genes from low to high, and the final rank value for each gene was used for further analysis.

**Figure 3:**
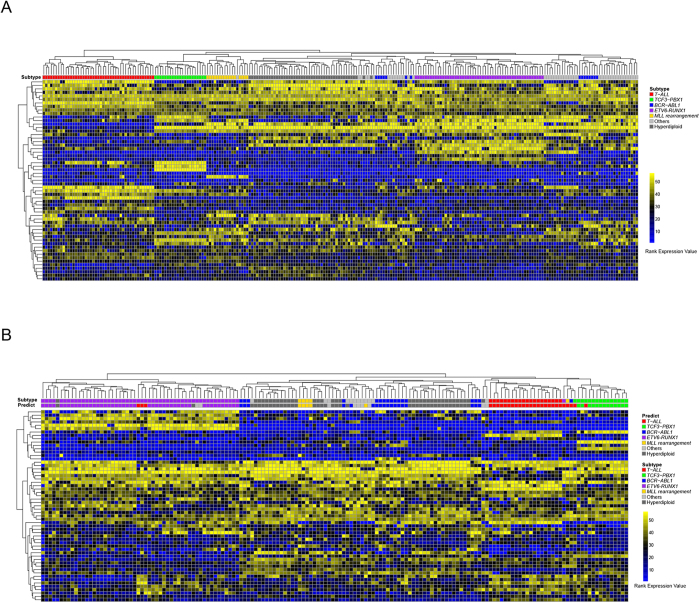
Hierarchical cluster of 240 microarray samples and 160 AFA samples. (**A**) Heatmap of 240 microarray samples. Expression levels of 57 marker genes were ranked from low to high in each sample. A high rank value represents a high expression value. The top color bar in the heatmap indicates the subtype each sample belongs to. (**B**) Heatmap of 160 AFA samples. The rank value was used as in (**A**). For the top color bar in the heatmap, the subtype bar indicates the real subtype for each sample, and the predict bar indicates the prediction results for each sample.

Microarray platforms have been widely used to study the classification of pediatric ALL patients. To test the performance of the 57 marker genes’ rank expression values in different platforms, we performed hierarchical clustering on the 240 microarray samples downloaded from the GEO data base (GSE28497) and the 160 AFA samples together using the within-sample rank values of the common 57 marker genes. High expression values were represented by high rank values. This non-supervised analysis readily segregates samples of different subtypes into different clusters for either the microarray or AFA samples, separately or in combination (Fig. 3A,B and Supplementary Figure S1). The microarray data are from Caucasian patients, and the AFA data are from Chinese patients, which demonstrate that the expression rank values of the 57 marker genes can be used to distinguish different subtypes even across different platforms and different ethnic groups.

### 57-marker gene-based SVM classifier construction based on microarray dataset

Although hierarchical clustering can distinguish samples of different subtypes according to the expression patterns of the 57 marker genes, it cannot predict the subtype of an individual sample without referencing the other background samples. Therefore, we utilized the SVM machine learning method to train a classification model. Because the number of AFA samples was too small to accomplish training and testing, we constructed the SVM model based on 240 published ALL microarray data^19^ and tested it on the 160 independent AFA samples.

Ten-fold cross validation was used to select the best C and Gamma for the SVM model. The total accuracy for the 10-fold cross validation results was 98.75%. Then, we applied this SVM classifier to the 160 AFA samples. As shown in Table 1, for each of the subtypes, the prediction accuracy and specificity were greater than 94%. These findings indicated that the classifier performed well for the AFA data. The sensitivity for the MLL rearrangement subtype was 80%. There were 5 patients with *MLL* rearrangement in total, and only 1 of them was predicted to be the T-ALL subtype. Figure 1B shows that this mis-predicted sample has a distinct gene expression profile compared with the other 4 *MLL* rearrangement samples but similar to the T-ALL subtype. Additionally, we observed that the prediction sensitivity for the “Others” subtype was only 50%, similar to the value found in previous studies. Although no fusion genes are detected in the “Others” subtype, some may have gene expression profiles similar to those of other known subtypes, such as *BCR-ABL1*. A recent study has defined *BCR-ABL1*-like subtypes in the “Others” samples based on the similarity of their gene expression profiles to *BCR-ABL1*-positive samples^10^. In our study, two of the “Others” samples were clustered together with *BCR-ABL1* samples and were predicted to be of the *BCR-ABL1* subtype, which indicates that they may belong to the *BCR-ABL1*-like subtype. For the other subtypes, the prediction sensitivity was very high, especially for *BCR-ABL1* (94.12%), T-ALL (100%) and hyperdiploid >50 (97.3%).

To test the classifier’s robustness, we also applied it to two other independent data sets that were based on microarray platforms. There are 190 patients in the COALL cohort and 107 patients in the DCOG cohort. By using the same rank expression values of the 57 marker genes, we obtained greater than 93% accuracy for each subtype except for the “Others” subtype (Supplementary Table S4, S5), with some belonging to the *BCR-ABL1*-like subtype. This finding indicates that our classifier is robust across different platforms.

## Discussion

Current treatment protocols for ALL patients emphasize risk-based therapy to reduce toxicity in low-risk patients and to also ensure appropriate and more aggressive therapy for those with a high risk of disease relapse. The accurate diagnostic classification of pediatric ALL is the initial step in deciding on an appropriate therapy strategy. For example, patients with an *ETV6-RUNX1* fusion gene usually have favorable long-term remission and survival rates; therefore, low-risk chemotherapy strategies are required. Patients with a *BCR-ABL1* fusion gene or an *MLL* rearrangement are in a high-risk group because of the high leukemic burdens and poorer prognosis^20^. Nevertheless, relapsed events usually occur in the patients with favorable subtypes, whereas some patients in the high-risk group can obtain long-term survival, which further emphasize the importance of molecular classification in ALL patients. More importantly, nearly half of ALL patients have no chromosomal translocation or fusion gene, which conceals the potential characteristics of these patients at the gene level. The lack of genetic congruence in ALL patients explains why different therapeutic effects are obtained among patients with the same cytogenetic and molecular biological disorders. Consequently, precise subtype classification at the initial diagnosis is critical for personalized therapy that avoids unnecessary over- or under-treatment.

AFA technology combines RT-PCR and capillary electrophoresis to deliver a simple and effective multiplex assay. Each chimeric primer contains a gene-specific sequence at the 3′ end and a universal tag sequence at the 5′ end. The adoption of universal primers overcomes the potential bias of traditional multiplexed PCR amplification methods, maintains the same amplification efficiency of each gene, and establishes a highly quantitative and sensitive assay. The universal priming strategy has been widely applied to the diagnosis, classification and prognostic evaluation of human cancers^21^,^22^,^23^,^24^.

Compared to the complex procedures involved in microarray-based detection methods, AFA assays only require three multiplexed RT-PCR experiments to obtain the gene expression profiles. Most importantly, only half a day is required for ALL patients to obtain their gene classification results at the cost of 100 US dollars, compared with nearly half a month and 500 US dollars in microarray assays. These time and cost savings can lead to better patient outcomes. The SVM classifier we constructed is based on microarray data, but it also performs well on AFA samples. This finding demonstrates that our multiplexed assays are convincing and reliable. The classifier provides a convenient and easy way to predict the subtype of clinical samples with high accuracy and specificity based on the AFA data of 57 marker genes. Additionally, the prediction results produced by the classifier can provide an important clue for clinical treatment. For example, for a patient with a known chromosomal translocation or fusion gene, the prediction results from this classifier may call for extra attention to monitor the effects of therapy.

In conclusion, this study describes the development of a novel multiplexed RT-PCR-based assay called AFA, which can detect pediatric ALL patients’ subtype within a half day at low cost and with high accuracy. The AFA assay offers a powerful diagnostic tool for rapid classification using a minimal amount of sample. Physicians are better able to provide appropriate clinical care with the rapid and accurate classification of the subtypes in pediatric ALL. However, it should be noted that if more samples and AFA datasets are included, the accuracy of the results will be higher. In the future, when enough AFA samples are collected, we can construct prediction models based on these AFA data, which will further improve the prediction accuracy and sensitivity of the assay.

## Additional Information

**How to cite this article**: Zhang, H. *et al*. An advanced fragment analysis-based individualized subtype classification of pediatric acute lymphoblastic leukemia. *Sci. Rep*. **5**, 12435; doi: 10.1038/srep12435 (2015).

## Supplementary Material

Supplementary Information

## Figures and Tables

**Table 1:** Prediction results for 160 AFA samples.

Subtype	TP	FP	TN	FN	Accuracy	Sensitivity	Specificity
*BCR-ABL1*	16	2	141	1	98.13%	94.12%	98.60%
*TCF3-PBX1*	13	0	145	2	98.75%	86.67%	100.00%
*MLL* rearrangement	4	1	154	1	98.75%	80.00%	99.35%
T-ALL	20	8	132	0	95.00%	100.00%	94.29%
*ETV6-RUNX1*	47	0	106	7	95.63%	87.04%	100.00%
Hyperdiploid > 50	36	4	119	1	96.88%	97.30%	96.75%
Others	6	3	145	6	94.38%	50.00%	97.97%

TP: True Positive; FP: False Positive; TN: True Negative; FN: False Negative.

Accuracy = (TP + TN)/(TP + FN + TN + FP); Sensitivity = TP/(TP + FN); Specificity = TN/(TN + FP).
