# Supplementary material for: An advanced fragment analysis-based individualized subtype classification of pediatric acute lymphoblastic leukemia
Source: Sci Rep. 2015 Jul 21;5:12435. doi: 10.1038/srep12435 (PMC4508914; doi:10.1038/srep12435)
Supplement: Supplementary Information [file srep12435-s1.pdf]

An Advanced Fragment Analysis-Based Individualized Subtype Classification of Pediatric Acute Lymphoblastic Leukemia

Han Zhang, Hao Cheng, Qingqing Wang, Xianping Zeng, Yanfen Chen, Jin Yan, Yanran Sun, Xiaoxi Zhao, Weijing Li, Chao Gao, Wenyu Gong, Bei Li, Ruidong Zhang, Li Nan, Yong Wu, Shilai Bao, Jing-Dong J. Han, and Huyong Zheng

Supplementary Methods

Bone marrow samples and total RNA isolation

The bone marrow samples were collected in ethylene-diaminetetraacetic acid (EDTA) tubes. Mononuclear cells were isolated from the diagnostic BM samples by Ficoll gradient centrifugation (MD Pacific, Tianjin, China; density: 1.077 g/ml). Total RNA was extracted using Trizol according to the manufacturer's instructions (Invitrogen, Paisley, UK) and was cryo-preserved in a -80°C freezer until use.

Mixture of RNA samples from 14 ALL patients

Fourteen RNA samples from 14 ALL patients of 7 different subtypes were randomly divided into 7 groups. Two samples within a group were mixed up into 100 ul with the final concentration of 50 ng/μl. 700 μl RNA mixture (50 ng/μl) was made consisting of 100 μl RNA in each group from No.1 tube to No.7 tube (*Supplementary Table S3*). The RNA mixture was diluted according to the different requirements.

AFA-based multiple assay optimization

To optimize the reaction, we used a mixture of RNA extracted from 14 ALL samples of 7 different subtypes, which was diluted to 20 ng/μl. The AFA-based multiplex assay was initially optimized using this mixture to test individual primer pairs in a single-plex assay to ensure a single amplicon of the correct size was generated for each target gene. Control reactions with no template and no reverse-transcriptase were conducted to ensure the absence of non-specific amplification products. Subsequent optimization of the incorporated multiplex primer pairs was conducted on the RNA mixtures to characterize the primer products obtained in multiplex reactions. Briefly, reverse primers of all gene targets and reference genes were mixed together to create a reverse-transcription (RT) primer pool (RT primer mix) at a final concentration of 500 nM that was used in the reverse transcription. All forward primers were mixed together to make a PCR primer pool (PCR primer mix) at a final concentration of 200 nM that was used in the PCR reactions. After the first round of multiplex PCR and capillary electrophoresis, a signal peak of moderate height was selected as a reference. Higher signal peaks were lowered by reducing the corresponding concentrations of reverse primers, and lower signal peaks were raised by increasing the primer concentration until all signal peaks were at moderate height.

Standard curve generation of AFA assays

The amplification efficiency of each of the primer pairs was diverse because multiple primers were incorporated into one reaction for the multiplexed assay. To represent the real expression levels of gene targets in the sample, a relative standard curve was

necessary for the relative quantitation of gene expression. Total RNA extracted from 14 ALL samples of 6 different subtypes were mixed together as the standard, which was serially diluted to 200 ng/μl, 100 ng/μl, 50 ng/μl, 25 ng/μl, 12.5 ng/μl, 6.25 ng/μl, 3.125 ng/μl and 1.56 ng/μl. The peak areas of each target gene and the endogenous reference controls were normalized to the peak area of the external reference *Kan*^{R}. Normalized data from the standard dilution series were used to generate a standard curve.

Supplementary Figure Legends

Supplementary Figure S1: Hierarchical cluster of the combined 160 AFA samples and 240 microarray samples.

Supplementary Figure S1

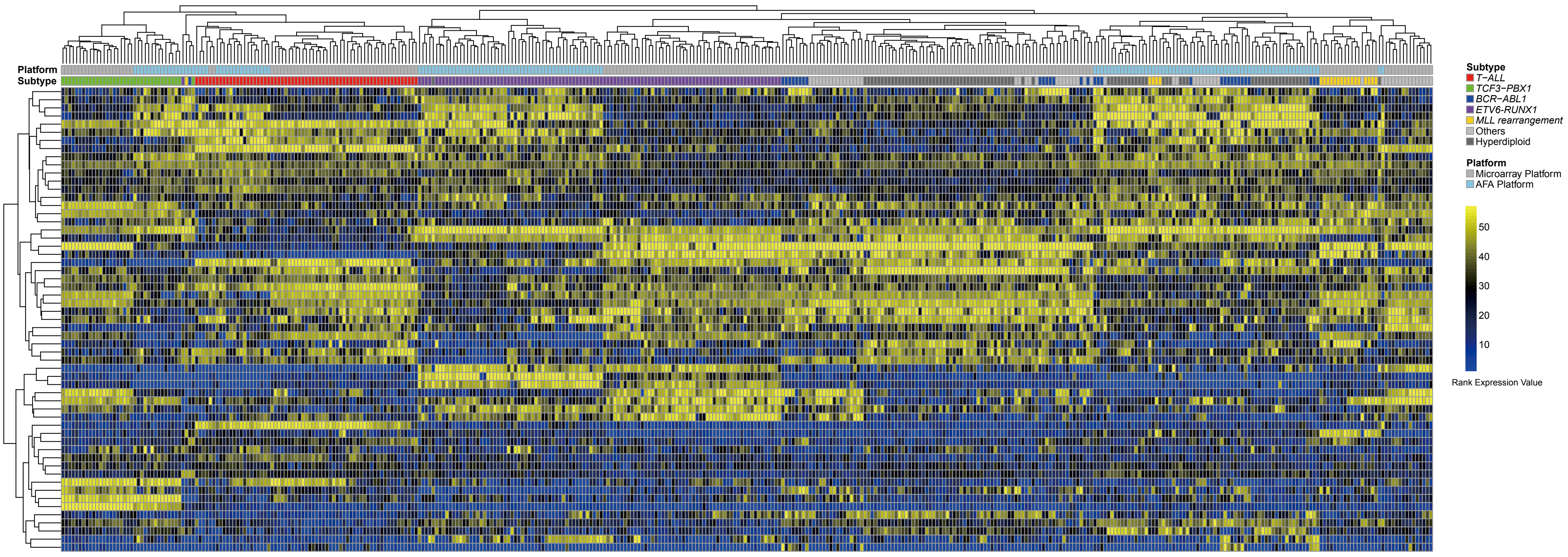

Supplementary Table S1

Forty-five fusion genes detected in Beijing Children's Hospital

No.	Fusion genes
1	*BCR-ABL1*
2	*ETV6-RUNX1*
3	*TCF3-PBX1*
4	*TCF3-HLF*
5	*SIL-TAL1*
6	*SET-CAN*
7	*MLL-AF4*
8	*MLL-ENL*
9	*MLL-AFX*
10	*MLL-AF1p*
11	*MLL-AF6*
12	*MLL-AF9*
13	*MLL-AF10*
14	*ETV6-ABL1*
15	*TLS-ERG*
16	*RUNX1-ETO*
17	*RUNX1-MDS1*
18	*PML-RARA*
19	*PLZF-RARA*
20	*CBFβ-MYH11*
21	*MLL-AF1q*
22	*MLL-MLL*
23	*MLL-AF17*
24	*MLL-ELL*
25	*DEK-CAN*
26	*NPM-ALK*
27	*PRKAR1A-RARa*
28	*STAT5b-RARa*
29	*NUMA1-RARa*
30	*NUP98-HoxAl1*
31	*NUP98-HoxA13*
32	*NUP98-HoxA9*
33	*NUP98-HoxC11*
34	*NUP98-HoxD13*
35	*NUP98-PMX1*
36	*RUNX1-EVI1*
37	*RUNX1-MTG16*
38	*ETV6-PDGFRA*
39	*FIP1L1-PDGFRA*
40	*FIP1L1-RARa*
41	*MLL-SEPT6*
42	*ETV6-JAK2*
43	*NPM-MLF1*
44	*ETV6-PDGFR*
45	*NPM-RARA*

Supplementary Table S2

Clinical features of the pediatric ALL cases for bone marrow samples

No.	Sex	Age of diagnosis (years)	WBC in PB at ID ($\times10^9$/L)	Percentage of blast cells in BM at ID (%)	Immunotype	Cytogenetic abnormality	Fusion gene	Outcome
1	M	7	144.8	86	T-ALL	—	—	Remission
2	M	12	29.8	97	c-B-ALL	t(1;19)(q23;p13)	*TCF3-PBX1*	Remission
3	M	5	70.5	83	c-B-ALL	t(1;19)(q23;p13)	*TCF3-PBX1*	Remission
4	M	8	59.1	72	T-ALL	—	—	Lost to followup
5	M	4	4.6	80	c-B-ALL	t(12;21)(p13;q22)	*ETV6-RUNX1*	Remission
6	M	6	3.9	96	c-B-ALL	t(12;21)(p13;q22)	*ETV6-RUNX1*	Remission
7	M	9	9.5	77.5	c-B-ALL	t(9;22)(q34;q11)(e1a2)	*BCR-ABL1*	Relapse
8	M	12	135.3	80	T-ALL	—	—	Remission
9	M	5	142.9	89.5	c-B-ALL	t(9;22)(q34;q11)(e1a2)	*BCR-ABL1*	Lost to followup
10	M	7	627.3	92	T-ALL	—	—	Remission
11	M	4	22.2	91	c-B-ALL	t(1;19)(q23;p13)	*TCF3-PBX1*	Remission
12	M	4	38	90	c-B-ALL	t(9;22)(q34;q11)(b2a2)	*BCR-ABL1*	Lost to followup
13	M	7	47.2	86.5	c-B-ALL	t(1;19)(q23;p13)	*TCF3-PBX1*	Remission
14	F	4	139.8	88	T-ALL	—	—	Remission
15	M	8	18.5	90	c-B-ALL	t(12;21)(p13;q22)	*ETV6-RUNX1*	Remission
16	M	5	48.8	98.5	c-B-ALL	t(12;21)(p13;q22)	*ETV6-RUNX1*	Remission
17	M	5	378.2	84	c-B-ALL	t(9;22)(q34;q11)(e1a2)	*BCR-ABL1*	Relapse
18	M	4	9.4	84	c-B-ALL	t(12;21)(p13;q22)	*ETV6-RUNX1*	Remission
19	F	11	37.78	94	c-B-ALL	t(1;19)(q23;p13)	*TCF3-PBX1*	Remission
20	M	10	454.78	82	T-ALL	—	—	Remission
21	M	7	22.3	82	T-ALL	—	—	Remission
22	F	4	27.96	91	c-B-ALL	t(12;21)(p13;q22)	*ETV6-RUNX1*	Remission
23	F	7	69.6	85	c-B-ALL	t(9;22)(q34;q11)(e1a2)	*BCR-ABL1*	Lost to followup
24	F	9	180	87	T-ALL	—	—	Relapse
25	F	4	5.7	94	c-B-ALL	t(12;21)(p13;q22)	*ETV6-RUNX1*	Remission
26	M	4	3.3	88.5	c-B-ALL	t(12;21)(p13;q22)	*ETV6-RUNX1*	Remission
27	M	8	5.1	95	T-ALL	—	—	Remission
28	F	2	3.7	80	c-B-ALL	t(12;21)(p13;q22)	*ETV6-RUNX1*	Remission

29	M	3	4.2	76	c-B-ALL	t(12;21)(p13;q22)	*ETV6-RUNX1*	Remission
30	M	10	61.7	87	T-ALL	—	—	Remission
31	M	4	13.8	81	c-B-ALL	t(1;19)(q23;p13)	*TCF3-PBX1*	Relapse
32	M	10	552.4	89	T-ALL	—	—	Remission
33	F	3	21.8	86	c-B-ALL	t(1;19)(q23;p13)	*TCF3-PBX1*	Remission
34	F	3	5.0	77.2	c-B-ALL	t(1;19)(q23;p13)	*TCF3-PBX1*	Remission
35	M	9	4.2	83	c-B-ALL	t(12;21)(p13;q22)	*ETV6-RUNX1*	Remission
36	M	7	24	94	T-ALL	—	—	Remission
37	F	2	102.6	91	c-B-ALL	t(12;21)(p13;q22)	*ETV6-RUNX1*	Remission
38	M	4	56.6	80	c-B-ALL	t(12;21)(p13;q22)	*ETV6-RUNX1*	Remission
39	M	8	85.8	90	T-ALL	—	—	Relapse
40	F	3	6.38	71	c-B-ALL	t(4;11)(q21;q23)(MLLex6-AF4(a:1414))	*MLL*	Lost to followup
41	M	5	3.76	73	c-B-ALL	t(1;19)(q23;p13)	*TCF3-PBX1*	Remission
42	M	4	4.71	83	c-B-ALL	t(12;21)(p13;q22)	*ETV6-RUNX1*	Remission
43	F	4	2.88	91	c-B-ALL	t(12;21)(p13;q22)	*ETV6-RUNX1*	Remission
44	M	4	5.51	87	c-B-ALL	t(12;21)(p13;q22)	*ETV6-RUNX1*	Remission
45	F	6	32.2	78	c-B-ALL	t(1;19)(q23;p13)	*TCF3-PBX1*	Remission
46	M	7	36.64	83	c-B-ALL	t(9;22)(q34;q11)(e1a2)	*BCR-ABL1*	Remission
47	M	9	87.53	91	c-B-ALL	t(9;22)(q34;q11)(e1a2)	*BCR-ABL1*	Lost to followup
48	M	9	79.24	90	c-B-ALL	t(9;22)(q34;q11)(b2a2)	*BCR-ABL1*	Dead
49	M	5	19.34	74	c-B-ALL	t(12;21)(p13;q22)	*ETV6-RUNX1*	Relapse
50	M	3	9.84	87	c-B-ALL	t(12;21)(p13;q22)	*ETV6-RUNX1*	Remission
51	M	3	7.02	86	c-B-ALL	t(12;21)(p13;q22)	*ETV6-RUNX1*	Remission
52	M	3	15.14	92	c-B-ALL	t(12;21)(p13;q22)	*ETV6-RUNX1*	Remission
53	M	4	9.8	67	c-B-ALL	t(12;21)(p13;q22)	*ETV6-RUNX1*	Remission
54	F	5	1.91	93	c-B-ALL	t(12;21)(p13;q22)	*ETV6-RUNX1*	Remission
55	F	5	284.87	93	T-ALL	—	—	Lost to followup
56	F	8	30.02	76	c-B-ALL	t(9;22)(q34;q11)(e1a2)	*BCR-ABL1*	Lost to followup
57	M	10	2.18	92	c-B-ALL	t(12;21)(p13;q22)	*ETV6-RUNX1*	Remission
58	M	10	249.61	85	c-B-ALL	t(9;22)(q34;q11)(e1a2)	*BCR-ABL1*	Lost to followup
59	M	4	—	91	c-B-ALL	t(12;21)(p13;q22)	*ETV6-RUNX1*	Remission
60	F	3	12.01	87	c-B-ALL	t(12;21)(p13;q22)	*ETV6-RUNX1*	Remission
61	F	5	18.29	94	pre-B-ALL	t(12;21)(p13;q22)	*ETV6-RUNX1*	Remission
62	F	7	2.03	71	pre-B-ALL	t(12;21)(p13;q22)	*ETV6-RUNX1*	Remission
63	M	11	148.47	93	T-ALL	del(1)(p34;p34)	*SIL-TAL1*	Remission
64	M	8	3.0	94	c-B-ALL	t(12;21)(p13;q22)	*ETV6-RUNX1*	Remission

65	F	5	6.86	69	c-B-ALL	hyperdiploid＞50	—	Remission
66	M	3	118.41	88	c-B-ALL	t(12;21)(p13;q22)	*ETV6-RUNX1*	Remission
67	F	2	2.61	89	c-B-ALL	t(12;21)(p13;q22)	*ETV6-RUNX1*	Remission
68	M	8	207.79	80	c-B-ALL	t(9;22)(q34;q11)(e1a2)	*BCR-ABL1*	Lost to followup
69	M	2	9.13	90	c-B-ALL	hyperdiploid＞50	—	Remission
70	M	3	—	19	pre-B-ALL	t(9;22)(q34;q11)(e1a2)	*BCR-ABL1*	Remission
71	M	1	9.57	88	c-B-ALL	hyperdiploid＞50	—	Remission
72	M	7	2.46	97	c-B-ALL	t(12;21)(p13;q22)	*ETV6-RUNX1*	Remission
73	M	10	7.83	48.5	c-B-ALL	t(1;19)(q23;p13)	*TCF3-PBX1*	Remission
74	M	4	17.80	77	c-B-ALL	hyperdiploid＞50	—	Remission
75	M	12	98.17	90	T-ALL	del(1)(p34;p34)	*SIL-TAL1*	Remission
76	F	6	8.0	94	c-B-ALL	hyperdiploid＞50	—	Remission
77	M	2	1.2	78	c-B-ALL	hyperdiploid＞50	—	Remission
78	M	4	35.0	87	pre-B-ALL	t(12;21)(p13;q22)	*ETV6-RUNX1*	Remission
79	M	6	25.10	93	pre-B-ALL	hyperdiploid＞50	—	Remission
80	M	2	6.04	70	c-B-ALL	hyperdiploid＞50	—	Remission
81	F	3	5.96	95	c-B-ALL	hyperdiploid＞50	—	Remission
82	F	7	31.60	88	pre-B-ALL	t(1;19)(q23;p13)	*TCF3-PBX1*	Remission
83	M	2	17.05	90	c-B-ALL	t(12;21)(p13;q22)	*ETV6-RUNX1*	Remission
84	M	7	58.62	93	c-B-ALL	t(9;22)(q34;q11)(e1a2)	*BCR-ABL1*	Remission
85	M	4	5.8	75	c-B-ALL	t(12;21)(p13;q22)	*ETV6-RUNX1*	Remission
86	M	1	36.14	91	pre-B-ALL	t(12;21)(p13;q22)	*ETV6-RUNX1*	Remission
87	F	3	14.65	71	c-B-ALL	t(9;22)(q34;q11)(e1a2)	*BCR-ABL1*	Remission
88	M	0.75	—	83	pre-B-ALL	diploid	—	Remission
89	M	5	10.50	80	c-B-ALL	hyperdiploid＞50	—	Remission
90	F	5	10.70	92	c-B-ALL	hyperdiploid＞50	—	Remission
91	M	7	27.56	87	c-B-ALL	t(12;21)(p13;q22)	*ETV6-RUNX1*	Remission
92	M	3	5.55	80	c-B-ALL	hyperdiploid＞50	—	Remission
93	M	9	—	—	c-B-ALL	t(12;21)(p13;q22)	*ETV6-RUNX1*	Remission
94	M	3	5.89	55	c-B-ALL	hyperdiploid＞50	—	Remission
95	M	2	5.83	90	c-B-ALL	t(12;21)(p13;q22)	*ETV6-RUNX1*	Remission
96	M	3	11.57	93	c-B-ALL	t(12;21)(p13;q22)	*ETV6-RUNX1*	Remission
97	F	1	5.60	96.5	c-B-ALL	hyperdiploid＞50	—	Remission
98	F	2	3.02	40	c-B-ALL	hyperdiploid＞50	—	Remission

99	M	4	12.68	75	c-B-ALL	hyperdiploid＞50	—	Remission
100	F	6	8.52	93	c-B-ALL	hyperdiploid＞50	—	Remission
101	M	11	64.30	95	T-ALL	—	—	Remission
102	M	4	7.53	85	pre-B-ALL	t(12;21)(p13;q22)	*ETV6-RUNX1*	Remission
103	F	1	42.13	91.5	pro-B-ALL	t(9;11)(p22;q23)(MLLex8/9-AF9)	*MLL*	Remission
104	F	9	423.81	100	T-ALL	—	—	Remission
105	M	5	7.78	91	c-B-ALL	t(12;21)(p13;q22)	*ETV6-RUNX1*	Remission
106	M	11	11.69	51	c-B-ALL	t(12;21)(p13;q22)	*ETV6-RUNX1*	Remission
107	F	2	2.49	91	c-B-ALL	hyperdiploid＞50	—	Remission
108	M	9	15.0	90	c-B-ALL	t(9;22)(q34;q11)(b3a2)	*BCR-ABL1*	Remission
109	F	3	7.67	97	c-B-ALL	hyperdiploid＞50	—	Remission
110	F	5	5.39	91	c-B-ALL	t(12;21)(p13;q22)	*ETV6-RUNX1*	Remission
111	F	3	3.78	80	c-B-ALL	hyperdiploid＞50	—	Lost to followup
112	M	4	26.41	90	c-B-ALL	hyperdiploid＞50	—	Remission
113	M	6	13.04	95	c-B-ALL	t(12;21)(p13;q22)	*ETV6-RUNX1*	Remission
114	M	4	4.38	87	c-B-ALL	t(12;21)(p13;q22)	*ETV6-RUNX1*	Remission
115	F	9	10.14	95.5	c-B-ALL	diploid	—	Remission
116	M	5	3.66	85	c-B-ALL	hyperdiploid＞50	—	Remission
117	F	11	5.56	87	c-B-ALL	diploid	—	Remission
118	M	8	254.67	91	c-B-ALL	diploid	—	Remission
119	F	5	7.15	96	c-B-ALL	t(12;21)(p13;q22)	*ETV6-RUNX1*	Remission
120	M	5	11.26	93	c-B-ALL	hyperdiploid＞50	—	Remission
121	M	2	17.73	95	c-B-ALL	t(12;21)(p13;q22)	*ETV6-RUNX1*	Remission
122	F	10	45.67	94.5	c-B-ALL	t(12;21)(p13;q22)	*ETV6-RUNX1*	Remission
123	M	10	287.10	91.5	T-ALL	—	—	Remission
124	M	3	44.22	94	c-B-ALL	t(1;19)(q23;p13)	*TCF3-PBX1*	Remission
125	M	1	2.70	78	c-B-ALL	hyperdiploid＞50	—	Remission
126	M	8	207.21	87	T-ALL	del(1)(p34;p34)	*SIL-TAL1*	Remission
127	F	7	14.80	87	c-B-ALL	t(1;19)(q23;p13)	*TCF3-PBX1*	Remission
128	F	0.83	3.66	46	pro-B-ALL	t(9;11)(p22;q23)(MLLex6/7-AF9)	*MLL*	Remission
129	F	6	2.30	94	c-B-ALL	hyperdiploid＞50	—	Remission
130	M	3	6.23	90	c-B-ALL	hyperdiploid＞50	—	Remission
131	M	2	—	—	c-B-ALL	t(12;21)(p13;q22)	*ETV6-RUNX1*	Remission
132	M	9	15.15	96	pro-B-ALL	t(1;11)(p32;q23)(MLLex7-AF1p)	*MLL*	Remission

133	F	5	37.4	95	c-B-ALL	hyperdiploid＞50	—	Remission
134	M	5	6.0	95	pro-B-ALL	diploid	—	Remission
135	M	5	8.03	93	c-B-ALL	hyperdiploid＞50	—	Remission
136	F	1	3.5	38	c-B-ALL	t(9;11)(p22;q23)(MLLex7/9-AF9)	*MLL*	Remission
137	M	4	54.44	92	c-B-ALL	t(9;22)(q34;q11)(b3a2)	*BCR-ABL1*	Remission
138	M	2	2.53	97.5	c-B-ALL	t(12;21)(p13;q22)	*ETV6-RUNX1*	Remission
139	M	13	50.99	93.5	c-B-ALL	diploid	—	Remission
140	M	3	8.24	96	c-B-ALL	hyperdiploid＞50	—	Remission
141	M	5	8.4	81	c-B-ALL	t(12;21)(p13;q22)	*ETV6-RUNX1*	Remission
142	M	3	6.3	97	c-B-ALL	hyperdiploid＞50	—	Remission
143	M	1	12.32	92	c-B-ALL	diploid	—	Remission
144	M	3	14.79	79	c-B-ALL	t(12;21)(p13;q22)	*ETV6-RUNX1*	Remission
145	M	2	2.97	90	c-B-ALL	hyperdiploid＞50	—	Remission
146	F	1	46.86	95	c-B-ALL	hyperdiploid＞50	—	Remission
147	M	2	27.44	95	c-B-ALL	t(12;21)(p13;q22)	*ETV6-RUNX1*	Remission
148	F	3	34.86	93.5	c-B-ALL	diploid	—	Remission
149	M	5	2.47	93.5	c-B-ALL	hyperdiploid＞50	—	Remission
150	M	4	8.57	96	m-B-ALL	diploid	—	Remission
151	M	3	8.92	16	c-B-ALL	diploid	—	Remission
152	F	4	2.95	92.5	c-B-ALL	hyperdiploid＞50	—	Remission
153	M	4	21.42	89	c-B-ALL	hyperdiploid＞50	—	Remission
154	F	8	1.7	33	c-B-ALL	hyperdiploid＞50	—	Remission
155	F	5	6.67	80	c-B-ALL	diploid	—	Remission
156	M	7	3.1	86	c-B-ALL	t(12;21)(p13;q22)	*ETV6-RUNX1*	Remission
157	M	11	5.58	81	c-B-ALL	t(9;22)(q34;q11)(e1a2)	*BCR-ABL1*	Remission
158	M	9	19.19	80	c-B-ALL	t(1;19)(q23;p13)	*TCF3-PBX1*	Remission
159	M	12	14.39	88.5	pre-B-ALL	diploid	—	Remission
160	F	4	8.55	83	c-B-ALL	hyperdiploid＞50	—	Remission

WBC, white blood cell; PB, peripheral blood; ID, initial diagnosis; BM, bone marrow; T-ALL, T lineage ALL; c-B-ALL, common B lineage ALL; pre-B-ALL, precursor B lineage ALL; pro-B-ALL, progenitor B lineage ALL; m-B-ALL, mature B lineage ALL.

Supplementary Table S3

Genes and primers used in the AFA multiplexed assay

PCR set	Entrez gene ID	Gene symbol	Forward primer	Reverse primer	RT primer mix (nM)	Size of product*
Set 1	8842	PROM1	TCTCCCTGTTGGTGATTTGT	CCAGTTTCCGACTCCTTTTG	62.5	139
	567	B2M	CCGTGTGAACCATGTGACTT	ATTCATCCAATCCAAATGCGG	20	144
	4778	NFE2	GAACTGACTTGGCAGGAGAT	GTGGTGGAGGTCCAAGGTAT	250	149
	7535	ZAP70	CGCTGCACAAGTTCCTGG	CGGTGCACAAAGTTCTTCTC	3000	155
	23089	PEG10	CTGAGACTCCATTTTGCTGC	CTCCTCTTCATGTCAGGCAA	62.5	159
	11119	BTN3A1	GAGCACAATGAAGCAAGAACA	AAGAGGGCCTTTTTCCATTCA	333.3	163
	1604	DAF †	GTGAAAATTCCTGGCGAGAAG	ATAAGGCTGTTTGAGGGATGC	62.5	169
	5087	PBX1	ACAACTCAGTGGAGCATTCA	CTTTGCTCTCGCAGGAGATT	1500	173
	22862	FNDC3A	ACGTGCAGCTAACAAAATGG	AAGGAACCTCCCAATTCACT	250	180
	10643	IMP-3	TCCACCGTAAAGAAAATGCG	TCAAGGGGATCTCTTCTGTG	250	184
	4192	MDK	CAAAGGCCAAAGCCAAGAAA	AGCTAACGAGCAGACAGAAG	41. 7	188
	969	CD69	CTCTTTGCATCCGGAGAGTG	GCCCACTGATAAGGCAATGA	125	194
	87	ACTN1	AAATCCAGACCCTAGCACG	GTGGGAGTTACACCATGCC	125	199
	3482	IGF2R	GACGGCTGCAATCAATGAAA	CCTGCTCTCTATGATGCACT	333.3	204
	7102	TSPAN7	CAGTTAATTGGCATGCTGCT	ATGTACTGTGCTCTAACATTCT	750	208
	4068	SH2D1A	GGCAAAATCAGCAGGGAAAC	CTCAGCACTCCAAGAACCTG	250	214
	—	pcDNA3.1(+)	CAGACAATCGGCTGCTCTGA	GCTTCAGTGACAACGTCGA	200	218
	50649	ARHGEF4	AGGAGGAAAAGTGAACCGAC	CTTCTCACCTGCTGGTTCTT	2000	224
	1293	COL6A3	CGTCCAACAGGTCATCTCTG	GTCAACCAGCCTCTCTATGA	2000	230
	6782	STCH †	ATTGAAGCTGCTAACCTTGC	ATTGTTTCCAGACATTGCTCG	2000	235
	5704	PSMC4	GGAAGACCATGTTGGCAAAG	TGAGCATCGAATCTCTTGGT	125	239
	26112	DKFZP434C171 †	AGGAGTCCATTCTGAGCCGA	TTCCTGTTGCAGGGTCGTAA	750	249
	2791	GNG11	AGTGAAGTTGCAGAGACAACA	AGCATGTTGGTTTTATCTGGG	2000	259
	2990	GUSB	GCTTCGAGGAGCAGTGGTA	TTCACCCACACGATGGCATA	2000	269
	—	Kan^R	CGGGAAAACAGCATTCCAGG	GTGACGACTGAATCCGGTGA	500	316

Set 2	4211	MEIS1	TTTTCACACTGGCCTTAAAGAG	GCCCCCGTAATGGGGTA	250	140
	567	B2M	CCGTGTGAACCATGTGACTT	ATTCATCCAATCCAAATGCGG	2	144
	7738	ZNF184	TCGGCTCCCTGCGTTA	AACACGCTGAGGTTGTCTAC	125	149
	22795	NID2	TTTCACAGCCCACATCTCTC	GACCATGTTTGGTTGATTGC	178.6	154
	29760	BLNK	ACGTGACCACTGGACAGTTA	AATATCATGGACCATCTTTTGAAGC	217.4	160
	5087	PBX1	ACTCAGTGGAGCATTCAGAT	CTCTCGCAGGAGATTCATCA	192.3	165
	5796	PTPRK	CCCATCCAAGTGGAATGTATG	AAGCCCATCCTAGGTACTGA	31.3	170
	7133	TNFRSF1B	ATGCCGGCTCAGAGAATAC	AGAGCTGGGTGTATGTGCT	73.5	180
	1687	DFNA5	CCTACTTCTTGGTCAGTGCC	GTTGGGTCTTCAAGATCAGATAC	178.6	185
	9467	SH3BP5	GGAGAAGAAACTCAAGAGAGC	TTCAGGGCCATCTTGTACTC	138.9	190
	55556	ENOSF1	GGCAGTGTCAATGAGAACCT	CTCACACACCCTATTTTCAAGG	1000	199
	9590	AKAP12	GATAAAGCGATCACACCCCA	CAGCAGCAGCATTCATTTTC	31.3	204
	3655	ITGA6	ACGCGGATCGAGTTTGATAA	CAAAGATGTCTCGGGATTCCT	125	209
	10602	CDC42EP3	CAGTAAGCCACCCACTAGACA	AGCCAGTACCAATAAGTTAACGTC	36.2	214
	—	pcDNA3.1(+)	CAGACAATCGGCTGCTCTGA	GCTTCAGTGACAACGTCGA	200	218
	51465	UBE2J1	AGATCATTACCATGCGCAGC	CTTGCCCACTTCAAATCGAC	26.9	224
	8503	PIK3R3	CGCAGAGAGGGGAATGAAAA	GATCTTTCGCAGCTGGATCA	1500	229
	5704	PSMC4	GGAAGACCATGTTGGCAAAG	TGAGCATCGAATCTCTTGGT	5	239
	23150	FRMD4B	GTCTTCTACAAATGCTTCTGGG	GTCTCTGCTCACTACTCTCC	250	249
	11027	LILRA2	AGCCACAATCACTCATCAGAGTA	GGGCATGGGAATGGGAGTTC	22.7	254
	1362	CPD	ACCGGATATTTGGTTTGCCA	GGAACTCATGGCTTAGGAGG	500	259
	2990	GUSB	GCTTCGAGGAGCAGTGGTA	TTCACCCACACGATGGCATA	166.7	269
	—	KanR	CGGGAAAACAGCATTCCAGG	GTGACGACTGAATCCGGTGA	500	316

	2195	FAT †	CCCCTTTGGTTTGACATCAC	GTTCTGCATCAAGAGGTTTGG	20	137
	567	B2M	CCGTGTGAACCATGTGACTT	ATTCATCCAATCCAAATGCGG	5	144
	2770	GNAI1	CAGAACTAGAGTGAAAACTACAGG	TGAATCCACTTCTTCCGCTC	200	151
	5101	PCDH9	GGCAACTCTGATCCCAACTC	CAGGAGGCATCCAGCAATTA	42	158
	240	ALOX5	TGCACATGTTCCAGTCTTCT	ACTGGTAGCCAAACATCAGG	9	163
	7249	TSC2	GACATCATCATCAAGGCGCT	AAAGTTCCTGTAGAGGTGCG	66	169
	4734	NEDD4	GCCAGACTCACCATTTTTGG	GATGAAGTAGGCAAAAGCACA	167	174
	150094	SNF1LK †	GAAGCTTCTGAACCATCCAC	CTCGTTCTCACTCAGGTGC	50	179
	1490	CTGF	GTTACCAATGACAACGCCTC	GCTCAAACTTGATAGGCTTGG	4.5	185
	663	BNIP2	AATTTGGCAGAACTAGCAGA	CACATTCTTCAGTCTTGTTTGGA	41	190
	9452	ITM2A	TCTGATGCCCCTCAATACTTC	AGATGCCAAGGTTACTAACATCA	83	195
	3385	ICAM3	TGACTGGCAACAGTCGGAT	TCCACTTGGCAGCGCA	500	200
Set 3	4082	MARCKS	GTTTCCCCTCTTGGATCTGT	TACCTTCACGTGGCCATTC	500	206
	9934	P2RY14	CTTCACTGAAAAGAGACCTCA	GCAATGAAGACCATACAGTACA	2500	211
	—	pcDNA3.1(+)	CAGACAATCGGCTGCTCTGA	GCTTCAGTGACAACGTCGA	200	218
	5366	PMAIP1	GCTCTGTAGCTGAGTGGG	AGAAGAGTTTGGATATCAGATTCAG	1500	223
	5937	RBMS1	GTGTTGGCTTTGCTAGGATG	ATGCCATGGTCTTCCATTAGG	1500	228
	27245	AHDC1	CCTACAGGTACCCAGGCTTTA	GAGGGATCTTGGCGTTGGT	250	234
	5704	PSMC4	GGAAGACCATGTTGGCAAAG	TGAGCATCGAATCTCTTGGT	12.5	239
	8936	WASF1	AAAGAGGAAGCAGAAGCAGA	GATCCACGTATGTCTGAGGT	62.5	246
	219654	C10orf56 †	CAGCTATCTCAACAGCTTCTTC	TTTGTTGAAGCACAGGTGGC	2500	251
	53405	CLIC5	AAAAATTAAACACCGCCCTGA	ACAGTTGCCGATGCTTTCT	100	257
	3512	IGJ	AACCATTTGCTTTTCTGGGG	TGAGGTGGGATCAGAGATATT	167	262
	2990	GUSB	GCTTCGAGGAGCAGTGGTA	TTCACCCACACGATGGCATA	750	269
	—	KanR	CGGGAAAACAGCATTCCAGG	GTGACGACTGAATCCGGTGA	500	316

*Total size of PCR product after adding the universal tag sequences. † The old gene symbols of DAF, STCH, DKFZP434C171, FAT, SNF1LK and C10orF56 are updated to CD55, HSPA13, CCDC69, FAT1, SIK1 and ZCCHC24, respectively.

Supplementary Table S4

190 COALL patients' prediction results

Subtype	TP	FP	TN	FN	Accuracy	Sensitivity	Specificity
BCR-ABL1	3	6	179	2	95.79%	60.00%	96.76%
TCF3-PBX1	9	0	177	4	97.89%	69.23%	100.00%
MLL rearrangement	4	0	186	0	100.00%	100.00%	100.00%
T-ALL	36	0	154	0	100.00%	100.00%	100.00%
ETV6-RUNX1	44	5	141	0	97.37%	100.00%	96.58%
Hyperdiploid＞50	35	3	143	9	93.68%	79.55%	97.95%
Others	33	12	134	11	87.89%	75.00%	91.78%

Supplementary Table S5

107 DCOG patients' prediction results

Subtype	TP	FP	TN	FN	Accuracy	Sensitivity	Specificity
BCR-ABL1	1	7	99	0	93.46%	100.00%	93.40%
TCF3-PBX1	2	0	105	0	100.00%	100.00%	100.00%
MLL rearrangement	4	0	103	0	100.00%	100.00%	100.00%
T-ALL	15	0	92	0	100.00%	100.00%	100.00%
ETV6-RUNX1	24	3	80	0	97.20%	100.00%	96.39%
Hyperdiploid＞50	26	5	74	2	93.46%	92.86%	93.67%
Others	19	1	73	14	85.98%	57.58%	98.65%
